# Itching in Scarlet Fever

**Published:** 1892-08

**Authors:** 


Itching in Scarlet Fevers is not always agreeable, but it has never been supposed to be a favorable sign, yet St Philippe (*Rev. Mens. des Mal. de Lienf.*, February, 1890,) according to A. F. C., in *Archiv. Ped.*, in a paper presents the following conclusions:

1. Scarlatina is a disease which is often accompanied by itching.
2. This variety usually has a favorable prognosis.
3. The itching is due to the fact that the eruption is not intense, and the cutaneous lesions not very profound.

The best application for the relief of this itching, or almost any other for that matter, is the following:

R Campho-phenique.............................oz. ss
Albolene Unguent.............................oz jss
M. Sig. Apply night and morning.

Another advantage is, that in the direction of personal disinfection.

